# Unveiling the Burden of Interactions Among Clinical Risk Factors for 1-Year Mortality in Hospitalized Older Patients

**DOI:** 10.3389/fmed.2021.771115

**Published:** 2021-11-25

**Authors:** Fabrizia Lattanzio, Valentina Corigliano, Luca Soraci, Alessia Fumagalli, Graziano Onder, Stefano Volpato, Antonio Cherubini, Carmelinda Ruggiero, Annalisa Cozza, Francesco Guarasci, Andrea Corsonello

**Affiliations:** ^1^Scientific Direction, Istituto Di Ricovero e Cura a Carattere Scientifico Italian National Research Centers on Aging, Ancona, Italy; ^2^Department of Clinical and Experimental Medicine, University of Messina, Messina, Italy; ^3^Unit of Geriatric Medicine, Istituto Di Ricovero e Cura a Carattere Scientifico Italian National Research Centers on Aging, Cosenza, Italy; ^4^Respiratory Unit, Istituto Di Ricovero e Cura a Carattere Scientifico Italian National Research Centers on Aging, Casatenovo, Italy; ^5^Department of Cardiovascular, Endocrine-Metabolic Diseases and Aging, IstitutoSuperiore di Sanitá, Rome, Italy; ^6^Department of Medical Sciences, University of Ferrara, Ferrara, Italy; ^7^Center for Clinical Epidemiology, School of Medicine, University of Ferrara, Ferrara, Italy; ^8^Geriatria, Accettazionegeriatrica e Centro di Ricerca per l'Invecchiamento, Istituto Di Ricovero e Cura a Carattere Scientifico Italian National Research Centers on Aging, Ancona, Italy; ^9^Orthogeriatric and Geriatric Units, Gerontology and Geriatric Sections, Department of Medicine and Surgery, University of Perugia, Perugia, Italy; ^10^Unit of Geriatric Pharmacoepidemiology and Biostatistics, Istituto Di Ricovero e Cura a Carattere Scientifico Italian National Research Centers on Aging, Ancona, Italy

**Keywords:** hospitalized older patients, anticholinergic burden, functional impairment, cognitive impairment, handgrip, depression

## Abstract

**Background:** Hospitalized older patients are particularly exposed to adverse health outcomes.

**Objective:** In this study, we aimed at investigating the prognostic interactions between disability in basic activities of daily living (BADL), cognitive impairment, low handgrip strength, anticholinergic cognitive burden (ACB), and depression on 1-year mortality.

**Setting and Subjects:** Our series consisted of 503 older patients discharged from acute care hospitals.

**Methods:** Disability in at least one BADL, ACB, depression, cognitive impairment, and low handgrip strength was considered in the analysis. One-year mortality was investigated by Cox regression analysis and prognostic interactions among study variables were assessed by survival tree analysis.

**Results:** Basic activities of daily living disability, ACB, cognitive impairment, and low handgrip strength were significantly associated with 1-year mortality. Survival tree analysis showed that patients with BADL disability and high ACB carried the highest risk of poor survival [hazard ratio (HR): 16.48 (2.63–74.72)], followed by patients with BADL disability and low ACB (HR: 8.43, 95% CI: 1.85–38.87). Patients with cognitive impairment and no BADL disability were characterized by a lower but still significant risk of mortality (HR: 6.61, 95% CI: 1.51–28.97) and those with high ACB scores and good cognitive and functional performance (HR: 5.28, 95% CI: 1.13–24.55).

**Conclusion:** Basic activities of daily living dependency, cognitive impairment, and ACB score were the three main predictors of 1-year mortality among patients discharged from acute care hospitals; the interaction between BADL dependency and ACB score was

found to significantly affect survival. Early identification of such high-risk patients may help tailor targeted interventions to counteract their detrimental effects on prognosis.

## Introduction

Hospitalized older individuals represent a complex and extremely heterogeneous portion of the geriatric population, exposed to a constant burden associated with multimorbidity, polypharmacy, and acute diseases, which may affect the overall quality of life and prognosis (1, 2).

Several prospective studies have identified multiple predictors of increased risk of death in this population (3, 4). Dependency in performing basic activities of daily living (BADL) is recognized as a contributor to mortality and recurrent hospitalization among older patients with chronic diseases (5, 6). Cognitive impairment is a well-known predictor of poor outcomes in geriatric populations, in terms of mortality (7). Depressive symptoms also are highly prevalent among older hospitalized patients (8) and may exert relevant negative functional and prognostic implications (9). Low handgrip strength is a dynamic indicator of poor muscle function (10) and the main risk factor for diagnosing sarcopenia (11); it was shown to predict mortality, disability, and physical functioning (12). Finally, medication burden was also associated with increased mortality (13), and exposure to anticholinergic medications is an important predictor of poor outcomes among hospitalized older patients (14–17).

Interestingly, the above risk factors are known to affect each other. For example, BADL dependency may increase the risk of depression (18). Additionally, low handgrip strength may favor the development of further physical and cognitive disability (19, 20). Anticholinergic cognitive burden (ACB) was also found to predict both functional and cognitive impairment among older patients discharged from acute care hospitals (14). Moreover, ACB impact on mortality was greater among patients with low handgrip strength, depressive symptoms, and functional dependency. Finally, depression was found to predict functional impairment among both community-dwelling and hospitalized older patients (21, 22).

Despite this bulk of evidence, BADL dependency, ACB, depression, low handgrip or cognitive impairment have only been considered as individual potentially interacting variables in the former studies (14, 15, 17, 23, 24). For this reason, we aimed at providing a comprehensive investigation of the prognostic interactions involving all these risk factors simultaneously present in a population of older patients discharged from acute care hospitals, to identify the most clinically relevant combinations and improve prognostic stratification.

## Materials and Methods

This study used data from a multicenter prospective observational study carried out in seven geriatric and internal medicine wards of Italian acute care hospitals. All patients consecutively admitted to participating wards between June 2010 and May 2011 were asked to participate in the study. Exclusion criteria included age < 65 years and unwillingness to participate in the study. After obtaining written informed consent, all participants were assessed within the first 24 h from hospital admission and followed up until discharge. Study researchers collected information about demographic and clinical characteristics, cognitive and functional status, and medication intake before, during hospitalization, and at discharge. Medications were coded according to the Anatomical Therapeutic and Chemical classification (25). After discharge, patients were reassessed at 3, 6, and 12 months. All ethics committees at participating institutions approved the study.

Overall, 1,120 patients were enrolled in the study. Patients with incomplete baseline data (*N* = 3) and those who died during hospitalization (*N* = 39) were excluded from our analysis. Patients with missing data for cognitive impairment (*N* = 218), depression (*N* = 129), and handgrip (*N* = 100), and those with incomplete follow-up data (*N* = 131) were also excluded, leaving a final sample of 503 patients to be included in the analysis.

Patients excluded from the study were older (83.1 ± 7.3 vs. 79.4 ± 7.0; *p* < 0.001) and had higher prevalence of BADL disability (52.7 vs. 21.1%, *p* < 0.001), cognitive impairment (69.4 vs. 41.3%, *p* < 0.001), depression (46.1 vs. 35.0%, *p* = 0.005), and higher average ACB score (1.4 ± 1.4 vs. 1.2 ± 1.1, *p* < 0.001) compared to those included in the study.

### Exposure Variables

Comprehensive geriatric assessment and medication data were collected at the time of discharge. Cognitive impairment was defined as having age- and education-adjusted Mini-Mental State Examination < 24 (26); the number of BADL (27) was calculated at discharge, and an analytic variable was created to identify patients with dependency in at least 1 BADL. Depression was defined as having a 15-item Geriatric Depression Scale (28) score > 5. Exposure to anticholinergic medications was quantified by calculating the anticholinergic cognitive burden (ACB) score (29), which was chosen because it was externally validated (30) and considered more accurate in the evaluation of central anticholinergic burden compared with other tools (31). The exposure variable based on the calculation of ACB score at discharge was categorized as follows: low-medium anticholinergic burden (ACB = 0 or 1 and ACB = 0 for patients taking no anticholinergic medications) and high anticholinergic burden (ACB score 2 or more). Muscle strength was assessed by handgrip strength, measured by calibrated hand dynamometer (North Coast Hydraulic Hand Dynamometer, North Coast Medical Inc, Morgan Hill, CA, USA), as previously described (32). According to the revised European Working Group on Sarcopenia in Older People 2 criteria, low muscle strength was classified as handgrip < 27 kg in men and < 16 kg in women (11). Finally, the cumulative number of exposure variables (low handgrip, BADL disability, depression, cognitive impairment, and high ACB) was also calculated and included in the analysis.

### Outcome

The outcome of this study was 1-year mortality. Data on living status during follow-up were obtained by interviewing the patients and/or their formal and/or informal caregivers. The time from the day of the study enrollment through the day of death was used as the time to failure variable for the model. Survivors were censored at the end of the follow-up. About patients who died during the follow-up period, date and place of death were retrieved by relatives or caregivers. The municipal registers were consulted when neither patients or relatives nor caregivers could be contacted.

### Covariates

Age, sex, number of diseases at discharge, and number of medications prescribed at discharge were considered as potential confounders in the analysis. Selected diagnoses known to affect prognosis in older patients (hypertension, heart failure, coronary artery disease, atrial fibrillation, peripheral artery disease, diabetes mellitus, chronic obstructive pulmonary disease, chronic kidney disease, stroke, dementia, and cancer) were also included in the preliminary analysis. Finally, to account for the continuity of exposure to anticholinergic drugs during the first follow-up period after discharge, the ACB score at 3-month follow-up was also considered as a potential confounder in the analysis.

### Statistical Analysis

First, we compared survivors and patients who died during follow-up concerning study variables and covariates. Continuous variables were reported as mean ± SD. Categorical variables were expressed as several cases (percentage). To compare the characteristics of the patients, according to 1-year survival status, we used the Student's *t*-test for continuous variables and the chi-squared test for categorical ones.

The relative risk of 1-year mortality related to either single study risk factors (low handgrip, cognitive impairment, functional impairment, depression, and ACB score) or the cumulative number of risk factors was then investigated by Cox regression analysis. We fitted three different Cox regression models: model A, adjusted for age and sex; model B, adjusted for age, sex, number of medications, and number of diagnoses; and model C, adjusted for age, sex, number of medications, and individual diagnoses associated with mortality in the preliminary analysis (heart failure, hypertension, coronary artery disease, cancer, and atrial fibrillation) instead of several medications. Models B and C were also repeated after adjusting for ACB score at 3-month follow-up.

Risk factors significantly associated with mortality in adjusted models were further analyzed by Venn diagram to investigate their overlapping. Therefore, to improve the graphical representation of the predictive models, we fitted a survival tree model based on study risk factors significantly associated with mortality in Cox regression models. Survival trees represent peculiar non-parametric alternatives to (semi)parametric models; they are characterized by extreme flexibility that allows automatic detection of certain types of interactions without specifying them beforehand. In this study, the splitting criterion was based on a node deviance measure between a saturated model log-likelihood and a maximized log-likelihood as proposed by Leblanc and Crowley (33), with further node adjustment to simplify the graphical representation of the fitted survival tree. To assess the performance of the fitted survival tree, the leaf node membership was added as a categorical variable in a Cox regression model using the node with the best survival as the reference category. Bootstrap model validation (1,000 resamplings) was performed to limit the bias of the estimates. The accuracy performance of the model was assessed by calculating the C-index value and 95%CI for leaf node membership and compared with that of individual study risk factors or the cumulative number of risk factors.

We also planned to perform sensitivity analyses. To account for potential residual confounding, survival tree analysis was repeated including patients with available data for risk factors significantly associated with the outcome. C-index of leaf node membership was separately calculated for each survival tree. Finally, survival tree analysis was repeated by forcing into survival tree analysis risk factor(s) not significantly associated with mortality.

Analyses were performed using SPSS (version 26.0, SPSS, Chicago, IL, USA) and rpart (34) and partykit (35) packages of R (version 4.0.2, R Foundation for Statistical Computing, Vienna, Austria).

## Results

Baseline characteristics according to 1-year mortality are depicted in Table 1. Among the 503 study participants (mean age: 79.4 ± 7.0, 50.7% women), 69 patients (13.7%) died during the follow-up. Patients who died were older, malnourished, and had a higher number of medications and diagnoses, a greater prevalence of heart failure, coronary artery disease, cancer, and atrial fibrillation, and a lower prevalence of hypertension. Patients who died during the follow-up were also characterized by a higher prevalence of low handgrip, cognitive impairment, BADL disability, depression, and high anticholinergic burden. ACB medications prescribed at discharge according to 1-year mortality are reported in Supplementary Table 1.

**Table 1:** Demographic and clinical characteristics of discharged patients grouped by 1-year mortality.

	**All patients (*n* = 503)**	**Survivors** **(*n* = 434)**	**Died** **(*n* = 69)**	***P***
Age, mean (± SD)	79.4 ± 7.0	78.9 ± 7.0	82.8 ± 6.3	<0.001
Gender, F, *n* (%)	255 (50.7%)	225 (51.8%)	30 (43.5%)	0.24
Heart failure, *n* (%)	142 (28.2%)	109 (25.1%)	33 (47.8%)	<0.001
Hypertension, *n* (%)	399 (79.3%)	356 (82.0%)	43 (62.3%)	<0.001
CAD, *n* (%)	158 (31.4%)	127 (29.2%)	31 (44.9%)	0.013
PAD, *n* (%)	43 (8.5%)	37 (8.5%)	6 (8.7%)	0.989
COPD, *n* (%)	206 (40.9%)	171 (39.4%)	35 (50.7%)	0.10
CKD, *n* (%)	243 (50.2%)	204 (48.6%)	39 (60.9%)	0.09
Cancer, *n* (%)	70 (13.9%)	43 (9.9%)	27 (39.1%)	<0.001
Diabetes, *n* (%)	154 (30.6%)	132 (30.4%)	22 (31.9%)	0.92
Dementia, *n* (%)	53 (10.5%)	47 (10.8%)	6 (8.6%)	0.745
				
Cerebrovascular, *n* (%)	83 (16.5%)	70 (16.1%)	13 (18.8%)	0.697
Atrial fibrillation, *n* (%)	93 (18.5%)	72 (16.6%)	21 (30.4%)	0.010
Number of medications, mean (± SD)	7.62 ± 2.79	7.47 ± 2.71	8.51 ± 3.15	0.011
Number of comorbidities, mean (± SD)	5.3 ± 2.7	5.1 ± 2.6	6.2 ± 2.8	0.003
**Study risk factors**				
ACB score				<0.001
0-1	355 (70.6%)	319 (73.5%)	36 (52.2%)	
2 or more	148 (29.4%)	115 (26.5%)	33 (47.8%)	
BADL disability	106 (21.1%)	76 (17.5%)	30 (43.5%)	<0.001
Cognitive impairment	208 (41.3%)	167 (38.5%)	41 (59.4%)	0.002
Low handgrip strength	233 (46.3%)	191 (44.0%)	42 (60.9%)	0.01
Depression	176 (35.0%)	147 (33.9%)	29 (42.0%)	0.24

*p-value from t-test or χ^2^ test as appropriate. Data are expressed as mean ± SD or n and percentage as appropriate*.

*BADL, basic activities of daily living; CAD, coronary artery disease; PAD, peripheral artery disease; COPD, chronic obstructive pulmonary disease; CKD, chronic kidney disease*.

Cox regression analysis showed that all study risk factors but depression were associated with increased 1-year mortality in age- and sex-adjusted models, and similar findings were obtained in models B and C after adjusting for potential confounders (Table 2). Such results were confirmed after including ACB score at 3-month follow-up in fully adjusted models B and C; the corresponding figures (hazard ratio and 95% CI) for model C including ACB score at 3 months were 4.88 (2.60–9.16) for ACB score, 2.42 (1.48–3.98) for BADL disability, 1.87 (1.12–3.12) for cognitive impairment, 1.78 (1.03–3.01) for low handgrip strength, and 1.31 (0.76–2.26) for depression.

**Table 2:** The Cox regression analysis of study risk factors in relation to 1-year mortality.

	**Model A** **HR (95%CI)**	**Model B** **HR (95%CI)**	**Model C** **HR (95%CI)**
ACB score 0–1	–	–	–
ACB score 2 or more	2.17 (1.33–3.43)	1.66 (1.01–2.80)	1.61 (1.00–2.71)
Low handgrip	1.85 (1.03–3.33)	1.77 (1.02–3.18)	1.72 (1.01–3.20)
Cognitive impairment	1.94 (1.19–3.17)	1.90 (1.16–3.11)	2.01 (1.21–3.33)
BADL disability	2.95 (1.79–4.86)	2.75 (1.65–4.56)	2.50 (1.53–4.08)
Depression	1.33 (0.80–2.19)	1.26 (0.76–2.08)	1.31 (0.76–2.36)

*Model A: adjusted for age and gender; Model B: adjusted for age, sex, number of medications and number of diagnoses; and Model C: as for model B with heart failure, hypertension, coronary artery disease, cancer, and atrial fibrillation instead of number of diagnoses*.

*ACB, anticholinergic cognitive burden; BADL, basic activities of daily living; HR, hazard ratio*.

**Figure 1:**
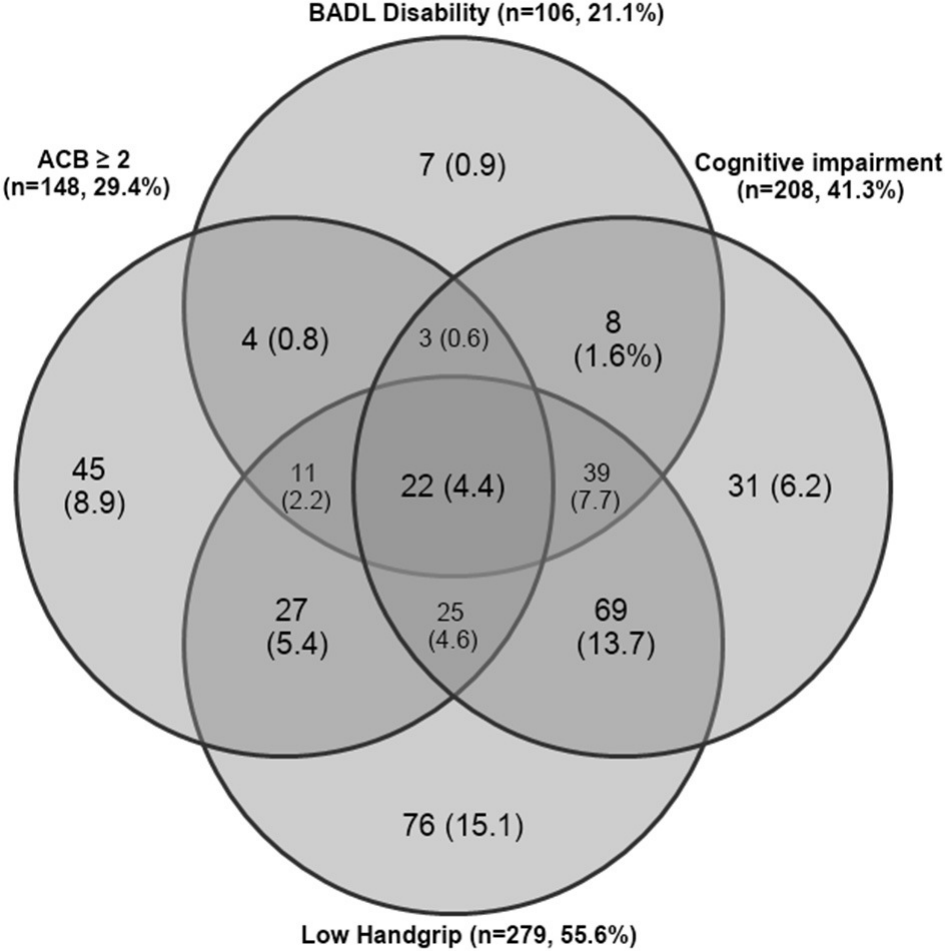
Venn diagram showing overlapping of study risk factors significantly associated with mortality in the study population.

Overlapping among study risk factors significantly associated with mortality is shown in Figure 1. Low handgrip strength was the most common risk factor in the study population (55.6%), followed by cognitive impairment (41.3%), high ACB (29.4%), and BADL disability (21.1%). In contrast to high ACB, low handgrip, and cognitive impairment, the presence of BADL disability was rarely observed alone and was often observed in combination with other risk factors. The most common combinations of study risk factors involved cognitive impairment and low handgrip strength (13.7%), BADL disability, cognitive impairment, and low handgrip strength (7.7%), low handgrip and high ACB (5.4%), and cognitive impairment, low handgrip, and high ACB (4.6%).

**Figure 2:**
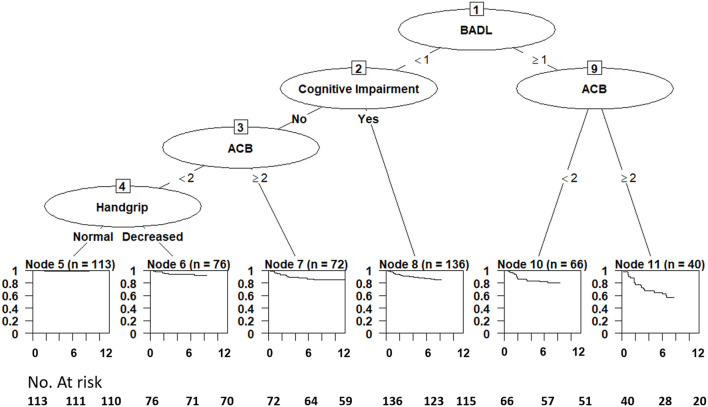
Survival tree of study risk factors showing 1-year mortality among patients carrying the leaf node combinations of study risk factors. ACB, anticholinergic cognitive burden; BADL, basic activities of daily living.

Results obtained by survival tree analysis are reported in Figure 2. Node 5 (patients with no BADL disability, no cognitive impairment, ACB score < 2, and normal handgrip, *n* = 113) showed the lowest mortality and was considered as the reference category in the Cox regression models. The highest risk of mortality was observed among patients belonging to Node 11 (BADL disability and ACB score of 2 or more) and Node 10 (BADL disability and ACB score < 2). Node 5 (no BADL disability, no cognitive impairment, ACB score < 2, and normal handgrip), and Node 6 (no BADL disability, no cognitive impairment, ACB score < 2, and impaired handgrip) shared the lowest mortality risk, while Nodes 7 and 8 showed intermediate mortality risk profiles. Descriptive features of leaf node groups are reported in Supplementary Table 2.

**Figure 3:**
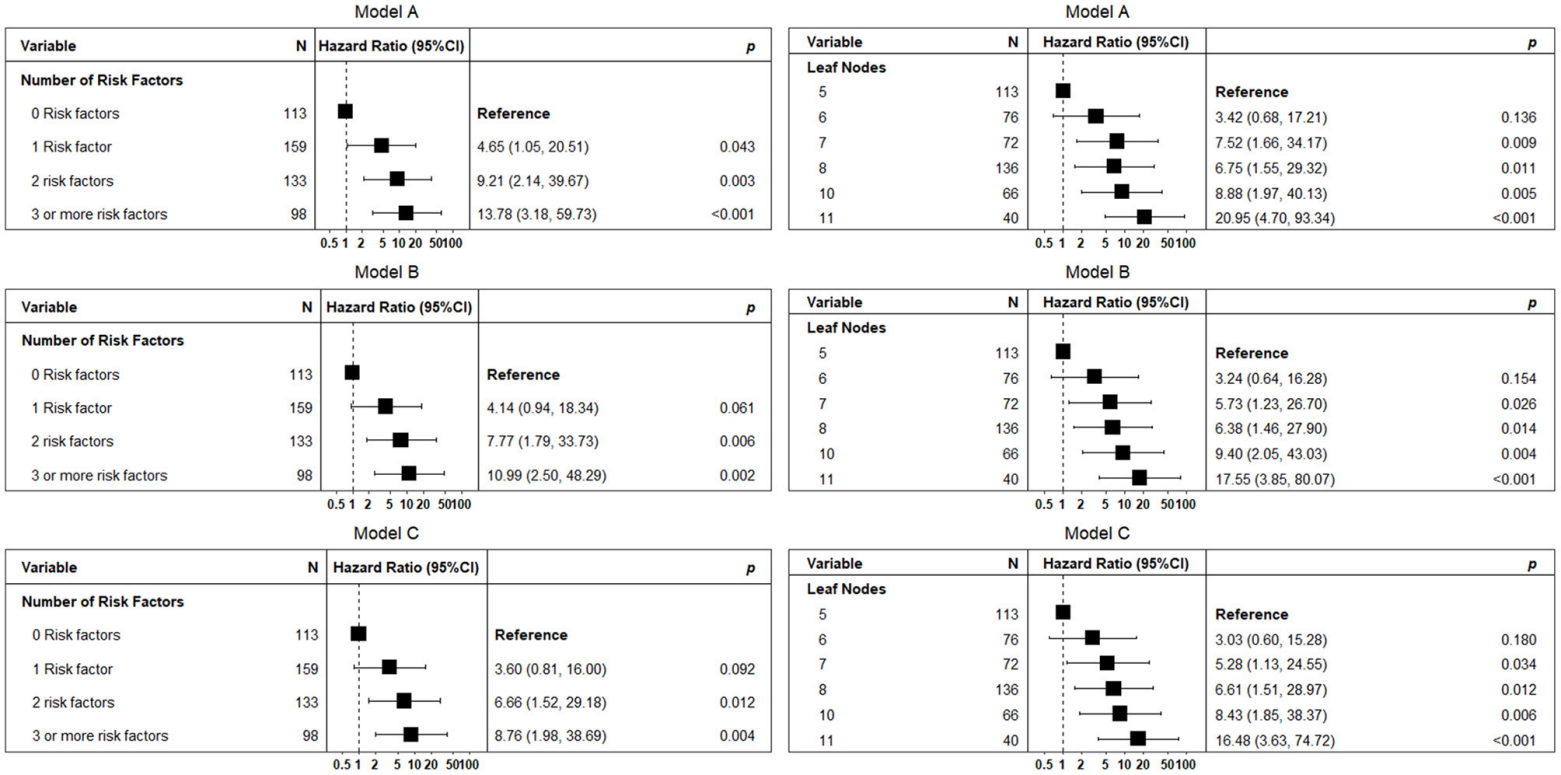
A forest plot showing the hazard ratio and 95% CIs associated with several selected risk factors (panel A) and leaf nodes (panel B), with time to 1-year mortality as the dependent variable. Squares represent the hazard ratio and the horizontal bars extend from the lower limit to the upper limit of the 95% CI of the estimate of the hazard ratio. Model A: adjusted for age and sex; Model B: adjusted for age, sex, and number of medications and diagnoses; and Model C: as for model B with heart failure, hypertension, coronary artery disease, cancer, and atrial fibrillation instead of several diagnoses.

The analysis including the number of risk factors or leaf node membership instead of single study risk factors showed that the coexistence of 2 or more study risk factors was significantly associated with mortality (Figure 3). Leaf node membership showed a progressive increase in mortality risk of death starting from Node 6.

The leaf node membership showed a good predictive accuracy (C-index: 0.812; 95% CI: 0.761–0.852), which was significantly better compared to that observed with several risk factors and individual risk factors (*p* < 0.05, see Table 3). Similar results were obtained by sensitivity analyses to fit a family of survival trees among patients with available BADL (*n* = 807), cognitive impairment (*n* = 670), handgrip (*n* = 621), and ACB score (*n* = 807) data (Supplementary Figure 1). The C-index of single survival trees was substantially comparable to that of the main survival tree (mean C-index 0.81, *p* = 0.85). Forcing the inclusion of depression in the survival tree analysis allowed us to identify a subgroup of patients with BADL disability and depression interacting in predicting 1-year mortality (Supplementary Figure 2).

**Table 3:** C-index in predicting 1-year mortality obtained by leaf nodes membership from survival tree analysis compared to individual risk factors or number of risk factors.

	**Age and sex-adjusted C-index**	***P*** **value**
Depression	0.661 (0.611–0.720)	<0.001
Low handgrip	0.685 (0.626–0.743)	<0.001
Cognitive impairment	0.701 (0.644–0.754)	<0.001
ACB score ≥ 2	0.699 (0.642–0.755)	<0.001
BADL disability	0.718 (0.661–0.774)	0.007
Number of risk factors	0.736 (0.685–0.787)	0.018
Leaf node membership	0.812 (0.761–0.852)	–

*ACB, anticholinergic cognitive burden; BADL, basic activities of daily living*.

## Discussion

In this prospective cohort study, we used a tree-based risk algorithm to identify the most relevant prognostic interactions among selected risk factors in older patients discharged from acute care hospitals. The survival tree analysis allowed the identification of selected combinations of risk factors leading to different prognostic outcomes concerning 1-year mortality. Patients with BADL disability and ACB score ≥ 2 (Node 11) were characterized by the highest mortality, followed by individuals with BADL disability and ACB score < 2 (node 10). Node 8 (cognitive impairment with no BADL disability) and Node 7 (ACB score of 2 or more with no cognitive impairment and no BADL disability) were characterized by a lower but still significantly increased mortality risk. Finally, cognitive impairment was highly prevalent among high-risk nodes but did not show any other significant interaction with either functional impairment or anticholinergic burden.

Functional impairment was an independent predictor of mortality in this population, thus confirming results from previous studies (36, 37); functional disability may be the expression of both presence and severity of multiple diseases on the health of individual and then characterize individuals at higher risk of poor long-term survival (38). Furthermore, dependency in at least one BADL showed a significant interaction with high ACB.

Patients with BADL disability and ACB of two or more were characterized by a 16-fold increased risk of mortality compared to the reference category, which confirms previous evidence regarding the need of moderating the use of anticholinergic medications in patients with functional impairment (17), even in the setting of this study considering a wider set of potential risk factors compared to the former one (17). The anticholinergic burden may, in fact, contribute to the effects of functional impairment on survival in older patients, by increasing the vulnerability of functionally impaired individuals to adverse drug reactions (39). Moreover, the anticholinergic burden has been shown to impair gait and balance and contribute to the risk of recurrent falls (40), representing a major cause of functional disability and death (14, 16). On the other side, anticholinergic medications may impair physical performance and worsen BADL dependency in patients with pre-existing disabilities, which might exponentially increase the risk of death (41).

Interestingly, depression seems to play a minor prognostic role in this study. Its prognostic weight was detected only in a small proportion of individuals after performing sensitivity analyses. Nevertheless, when forcing depression in the survival tree we could intercept a subgroup of patients with BADL disability and low ACB in which depression played an adjunctive negative prognostic role. At variance, the lack of interaction between BADL disability and either cognitive impairment or handgrip strength suggests that all these risk factors may impact prognosis through independent pathways.

Among patients with BADL independence, cognitive impairment was significantly associated with the outcome (Node 8), thus confirming previous evidence regarding this association, even in patients without diagnosis of dementia (42, 43); possible explanations for this association include poor adherence to medications, increased drug burden due to the treatment of cognitive disturbances, and increased incidence of frailty (43). Anticholinergic medications carried a less striking, but still important prognostic weight in patients with BADL independence; drugs with anticholinergic properties may increase the risk of cognitive impairment and dementia, falls and mortality (15, 17, 24). Furthermore, these drugs may have cardiovascular and neurological effects (44, 45), such as arrhythmias, syncope, hallucinations, and seizures, that might provoke serious adverse events, ultimately leading to death (46).

In the end, low handgrip strength, despite its high prevalence, did not independently contribute to the risk of mortality in the survival tree analysis, thus suggesting that its detrimental consequences are probably mediated by other factors.

The findings of this study may have several clinical and prognostic implications. The major prognostic interaction involved functional disability and ACB score, consequently, the assessment of functional status and revision of drug treatment during hospital stay should be important cornerstones of clinical evaluation of older hospitalized patients, to identify high-risk patients to whom reserve individualized pharmacological treatments. In this regard, the ACB score may represent a useful index of both pharmacological and mortality risk and may help predict future functional decline (14). A high ACB score was a significant predictor of mortality among patients with preserved functional and cognitive abilities; for this reason, careful and tailored prescribing and regular monitoring of anticholinergic medications should not be scotomized when dealing with these patients.

### Limitations

This study has some limitations. First, given the observational design, confounding by indication is a relevant limitation in the study. Even if results were confirmed in sensitivity analyses, a greater prevalence of depression, cognitive, and functional impairment, and a greater ACB score were found among patients excluded because of missing data. Additionally, missing may have contributed to the observation of a less relevant role of depression and handgrip in survival tree analyses. Second, the results of the survival tree analysis may lack the precision of estimates due to the small sample size, and wide CIs are in keeping with this hypothesis. Third, our results identify variables that by themselves may influence the outcome, and we could not account for illness severity, duration, and management of individual diagnoses. Fourth, the short duration of follow-up does not allow to optimally explore the long-term association between study risk factors and mortality. Additionally, our database included only limited information about the duration of exposure to ACB drugs and we could only calculate the ACB score at a 3-month follow-up. While adjusting the analysis for the ACB score at 3 months did not affect our main findings, we could not fully explore the effect of long-lasting exposure to anticholinergic medications compared with a shorter duration of treatment. Finally, the lack of information about post-acute care utilization did not allow us to investigate the impact of post-discharge care on study results. Finally, our results apply to the population of older patients discharged from acute care hospitals and cannot be generalized to the general older population.

On the other hand, the main strengths of our study are represented by the inclusion of a real-world unselected population of hospitalized older patients, the thorough evaluation of medications, and the use of a comprehensive geriatric assessment to explore the independent effect of several risk factors. In addition, this is the first study that identified several important interactions between previously investigated risk factors taken altogether by using the survival tree approach. Furthermore, the high stability and reproducibility of survival trees in sensitivity analyses and the high level of predictive accuracy estimates add further significance to study findings. If confirmed on larger studies, such interactions may have significant clinical and prognostic implications, which might suggest the need of using appropriate interventions to counteract the negative effects of selected risk factors on outcomes of patients. In this regard, hospital physicians should consider the assessment of ACB and functional and cognitive status as very important components of clinical practice.

### Conclusion and Implications

Functional impairment was the main predictor of 1-year mortality among hospitalized older patients and was found to interact with ACB score. Among patients with preserved functional capacity, cognitive impairment and anticholinergic burden were the main predictors of 1-year mortality. These findings support the importance of a careful evaluation of functional and cognitive status, and anticholinergic burden among older patients discharged from acute care hospitals to de-prescribe anticholinergic medications whenever possible, especially in patients carrying a selected combination of risk factors.

## Data Availability Statement

The datasets presented in this study can be found in online repositories. The names of the repository/repositories and accession number(s) can be found below: Data are available for CRIME study researchers at IRCCS INRCA (www.inrca.it).

## Ethics Statement

The studies involving human participants were reviewed and approved by Ethics Committee of the Catholic University of Rome (Project identification code: P/582/CE/2009). The patients/participants provided their written informed consent to participate in this study.

## Author Contributions

ACor, VC, LS, FG, ACoz, and AF participated in data analysis, manuscript writing, and revising and manuscript approval. GO, SV, CR, AChe, and ACor participated in data collection and writing, revising, and approving manuscript. FL participated in writing the manuscript, revising it for important intellectual content, and approval. All authors contributed to the article and approved the submitted version.

## Funding

This study was funded by Italian National Research Center on Aging (IRCCS INRCA) intramural research funds. The CRiteria to assess Inappropriate Medication use among Elderly complex patients (CRIME) project was partially supported by a grant from the Italian Ministry of Health (GR-2007 685638).

## Conflict of Interest

The authors declare that the research was conducted in the absence of any commercial or financial relationships that could be construed as a potential conflict of interest.

## Publisher's Note

All claims expressed in this article are solely those of the authors and do not necessarily represent those of their affiliated organizations, or those of the publisher, the editors and the reviewers. Any product that may be evaluated in this article, or claim that may be made by its manufacturer, is not guaranteed or endorsed by the publisher.
