# Supplementary material for: Unveiling the Burden of Interactions Among Clinical Risk Factors for 1-Year Mortality in Hospitalized Older Patients
Source: Front Med (Lausanne). 2021 Nov 25;8:771115. doi: 10.3389/fmed.2021.771115 (PMC8655869; doi:10.3389/fmed.2021.771115)
Supplement: Supplementary file 1 [file Data_Sheet_1.docx]

Supplementary Material

# Supplementary Figures and Tables

## Supplementary Figures

**
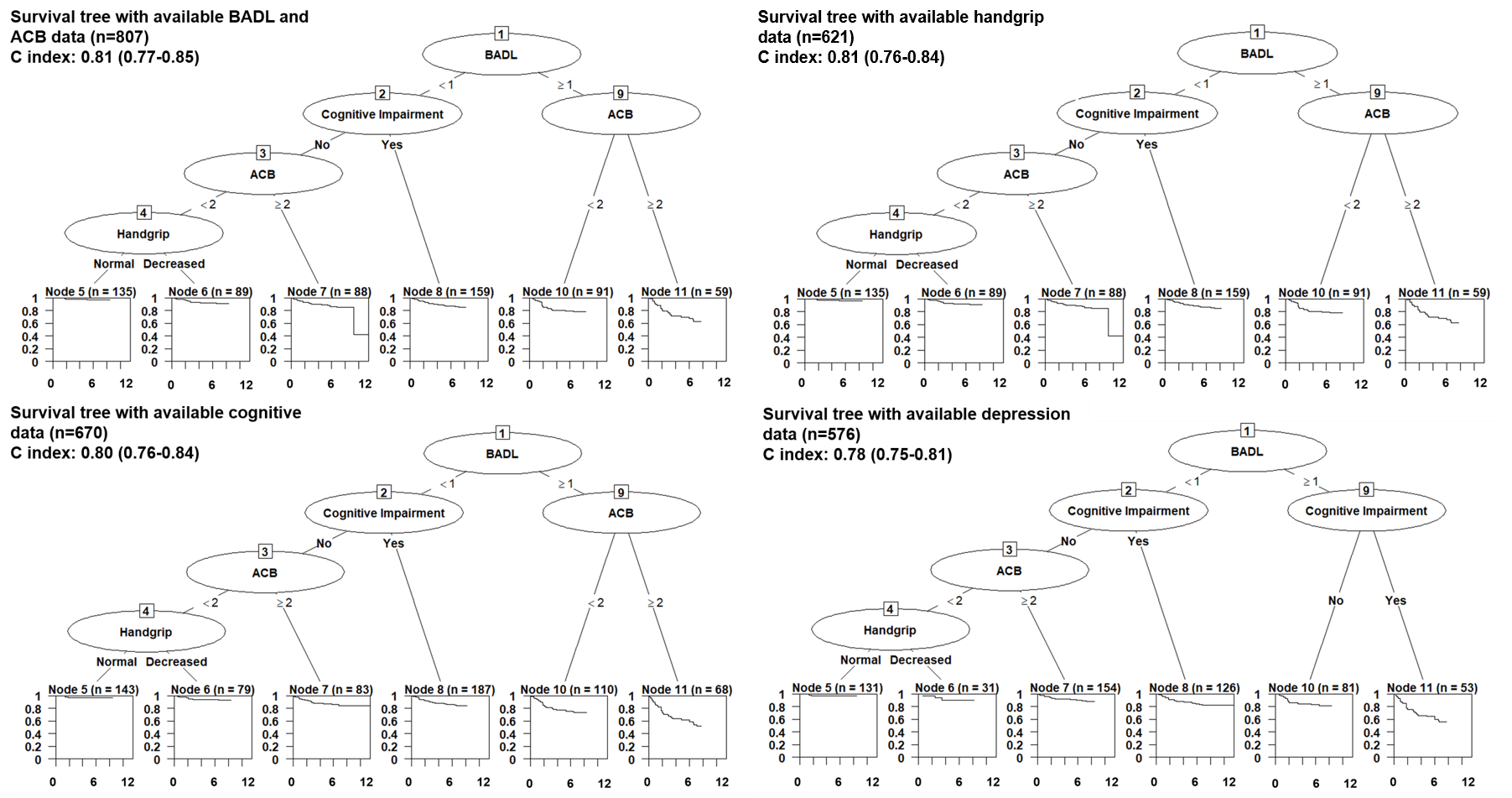
**

**Supplementary Figure 1. Survival trees obtained by performing sensitivity analysis on study results.**

**
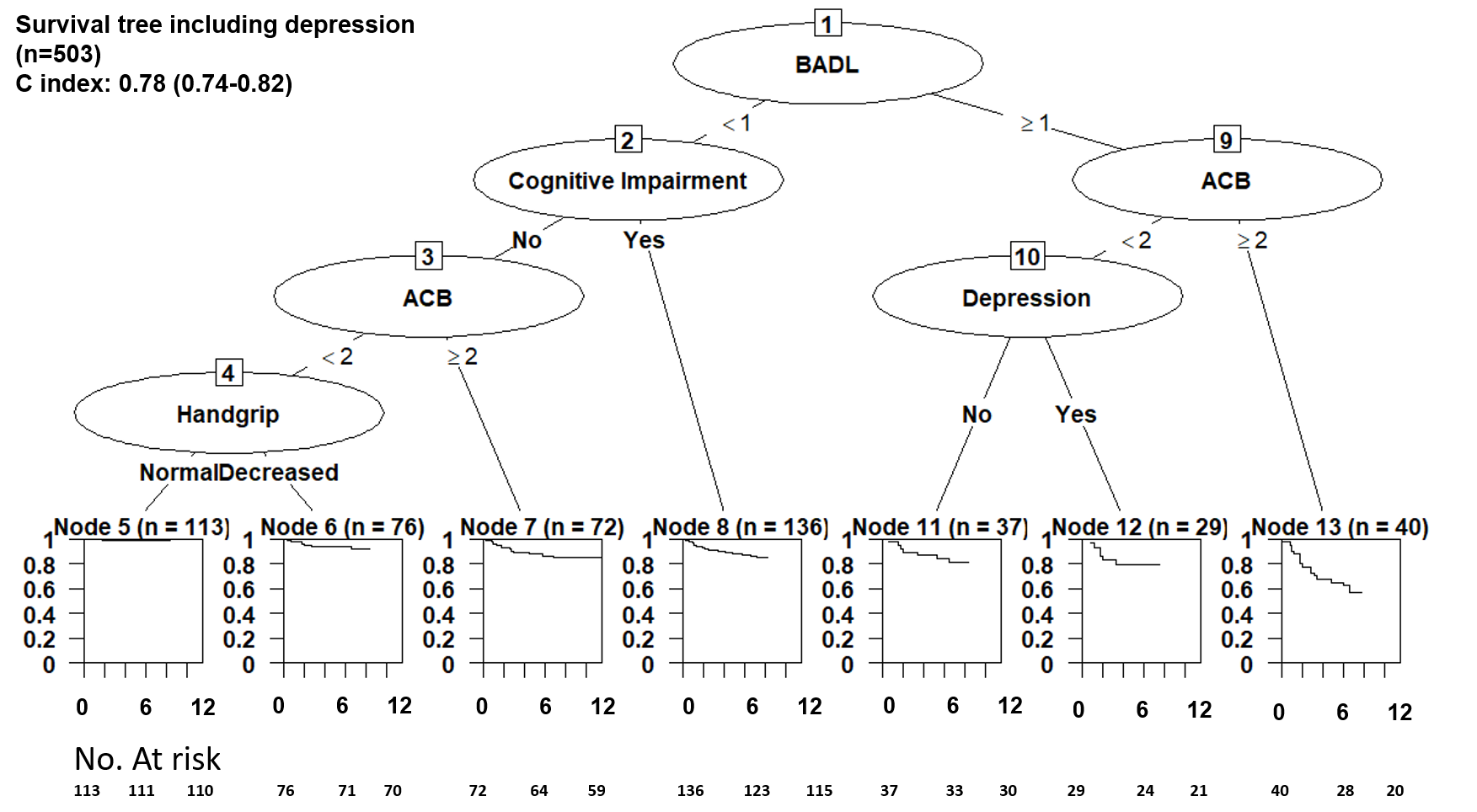
**

**Supplementary Figure 2. Survival tree obtained by forcing the presence of depression into the model.**

**1.2 Supplementary Tables**

**Supplementary Table 1. Anticholinergic Cognitive Burden (ACB) listed medications prescribed at discharge in the study population according to 1-year survival status.**

|  | **Survived (n=434)** | **Dead (n=69)** |
| --- | --- | --- |
| **ACB score 1** | Furosemide 198 (45.6%)  Prednisone 34 (7.8%)  Digoxin 25 (5.8%)  Isosorbide 23 (5.3%)  Codeine 17 (3.9%)  Warfarin 15 (3.5%)  Alprazolam 15 (3.5%)  Atenolol 14 (3.2%)  Nifedipine 12 (2.8%)  Ranitidine 9 (2.7%)  Trazodone 7 (1.6%)  Chlortalidone 3 (0.7%)  Cetirizine 3 (0.7%)  Haloperidol 3 (0.7%)  Diazepam 2 (0.5%)  Theophylline 2 (0.5%)  Colchicine 1 (0.2%)  Risperidone 1 (0.2%)  Fentanyl 1 (0.2%)  Captopril 1 (0.2%)  Aripiprazole 1 (0.2%)  Hydrocortisone 1 (0.2%) | Furosemide 51 (73.9%)  Prednisone 10 (14.5%)  Digoxin 7 (10.1%)  Isosorbide 6 (8.7%)  Codeine 4 (5.8%)  Warfarin 3 (4.4%)  Trazodone 3 (4.3%)  Atenolol 2 (2.9%)  Ranitidine 2 (2.9%)  Theophylline 1 (1.4%)  Fentanyl 1 (1.4%) |
| **ACB score 2** | Carbamazepine 2 (0.5%)  Oxcarbazepine 1 (0.2%) | Carbamazepine 1 (1.4%)  Meperidine 1 (1.4%) |
| **ACB score 3** | Quetiapine 8 (1.8%)  Amitryptiline 2 (0.5%)  Scopolamine 2 (0.5%)  Promazine 1 (0.2%)  Olanzapine 1 (0.2%)  Paroxetine 3 (0.7%) | Paroxetine 3 (4.3%)  Quetiapine 2 (2.9%) |

*Notes:* ACB=Anticholinergic Cognitive Burden.

**Supplementary Table 2. Characteristics of patients included in the study according to leaf node membership.**

|  | **Node 5 (n=113)** | **Node 6 (n=76)** | **Node 7 (n=72)** | **Node 8 (n=136)** | **Node 10**  **(n=66)** | **Node 11 (n=40)** | ***p trend*** |
| --- | --- | --- | --- | --- | --- | --- | --- |
| Age, mean ± SD | 74.7 ± 6.0 | 80.9 ± 6.7 | 79.5±5.4 | 80.4±7.3 | 82.1±5.7 | 82.2±7.6 | <0.001 |
| Gender, F, n(%) | 49 (43.4%) | 32 (42.1%) | 29 (40.3%) | 73 (53.7%) | 47 (71.2%) | 25 (62.5%) | 0.001 |
| Heart failure, n(%) | 12 (10.6%) | 15 (19.7%) | 36 (50.0%) | 36 (26.5%) | 22 (33.3%) | 20 (52.5%) | <0.001 |
| Hypertension, n(%) | 94 (83.2%) | 59 (77.6%) | 61 (84.7%) | 107 (78.7%) | 49 (74.2%) | 29 (72.5%) | <0.001 |
| Coronary artery disease, n(%) | 29 (25.7%) | 16 (21.0%) | 36 (50.0%) | 42 (30.9%) | 16 (24.2%) | 19 (47.5%) | 0.58 |
| Peripheral arterial disease, n(%) | 4 (3.5%) | 10 (13.1%) | 7 (9.7%) | 13 (9.6%) | 6 (9.1%) | 3 (7.5%) | 0.38 |
| Chronic obstructive pulmonary disease, n(%) | 37 (32.7%) | 31 (40.8%) | 36 (50.0%) | 61 (44.8%) | 22 (33.3%) | 19 (47.5%) | 0.15 |
| Chronic kidney disease, n(%) | 42 (38.9%) | 36 (48.0%) | 40 (56.3%) | 72 (55.0%) | 31 (50.8%) | 22 (57.9%) | 0.18 |
| Cancer, n(%) | 10 (8.8%) | 14 (18.4%) | 12 (16.7%) | 14 (10.3%) | 12 (18.2%) | 8 (20.0%) | 0.24 |
| Diabetes, n(%) | 32 (28.3%) | 20 (26.3%) | 22 (30.6%) | 47 (34.6%) | 15 (22.7%) | 18 (45.0%) | 0.25 |
| Dementia, n(%) | 2 (1.8%) | 2 (2.6%) | 2 (2.8%) | 24 (17.6%) | 16 (24.2%) | 7 (17.5%) | <0.001 |
| Cerebrovascular disease, n(%) | 14 (12.4%) | 7 (9.2%) | 3 (4.2%) | 26 (19.1%) | 18 (27.3%) | 15 (37.5%) | <0.001 |
| Atrial fibrillation, n(%) | 8 (7.1%) | 9 (11.8%) | 26 (36.1%) | 22 (16.2%) | 15 (22.7%) | 13 (32.5%) | <0.001 |
| Number of medications, mean ± SD | 6.3±2.6 | 7.2±2.6 | 9.3±2.4 | 7.6±2.8 | 7.2±2.8 | 8.9±2.3 | <0.001 |
| Number of comorbidities, mean ± SD | 4.2±2,4 | 5.0±2.2 | 6.3±3.1 | 5.1±2.4 | 5.9±2.7 | 6.7±2.7 | <0.001 |
| Anticholinergic burden of 2 or more, n(%) | 0 (0%) | 0 (0%) | 72 (100%) | 36 (26.5%) | 0 (0%) | 40 (100%) | <0.001 |
| Low Handgrip, n(%) | 0 (0%) | 76 (100.0%) | 27 (37.5%) | 92 (67.7%) | 51 (77.3%) | 33 (82.5%) | <0.001 |

Data are expressed as mean (±SD) or number (percentages).
